# Top ten causes of death in Malaysia for the years 2013 and 2019

**DOI:** 10.1016/j.lanwpc.2024.101066

**Published:** 2024-04-09

**Authors:** Wan-Fei Khaw, Heng Yaw Yong

**Affiliations:** a Institute for Public Health, National Institutes of Health, Ministry of Health Malaysia, Selangor, Malaysia; b Division of Nutrition and Dietetics, School of Health Sciences, International Medical University, Kuala Lumpur, Malaysia

## Introduction

Examining mortality rate trends, especially those influenced by leading causes of death, is essential for comprehensively evaluating societal health and stability. These trends encompass medical, social, and economic dimensions, providing policymakers with invaluable insights to shape effective future strategies. This study focuses on changes in deaths and age-adjusted mortality rates (AAMR) related to Malaysia's top ten causes of death for the years 2013 and 2019. The choice of 2013 as the starting point is deliberate, considering the transition in heart disease and stroke within Malaysia's causes of death during that period, as well as improved mortality estimation using cause-specific mortality fractions obtained from verbal autopsy data.^1^ This research fills a notable gap, as there is currently no published data on the causes of death in Malaysia, a middle-income country. The findings aim to inform evidence-based policymaking and contribute to a comprehensive understanding of health dynamics in the Malaysian population, facilitating targeted interventions for improved public health outcomes.

## Methods

The calculation of mortality in Malaysia utilized methodologies developed by Murray and Lopez for the Global Burden of Disease (GBD)^2^ and the specific methodology used in the Malaysian Burden of Disease detailed in the technical reports.^3^^,^^4^ Mortality data, including age, sex, cause of death and ICD-10 codes, from January 1 to December 31, 2013 and 2019 and the population counts were obtained from the Department of Statistics Malaysia (DOSM). Total deaths and age-adjusted mortality rates were computed through the direct method, employing the WHO standard population as a basis.

## Results

The Table 1 presents the leading causes of death in Malaysia for the years 2013 and 2019. In 2019, the top three causes of death were ischaemic heart disease, cerebrovascular diseases (CVD), and lower respiratory infection. There were notable changes in the numbers and ranking of deaths compared to 2013. Ischaemic heart disease was the top leading cause of death in 2019, with an estimate of 35,704 deaths, and was substantial increased from 2013 to 2019, the greatest increase in AAMR by 40.6%. Cerebrovascular disease shifted from the leading cause in 2013 to the second leading cause in 2019, with decline in AAMR by 13.7%. Lower respiratory infection was maintained as the third leading causes in 2013 and 2019, with AAMR slightly increased by 5.6%. Increases in AAMR due to colon and rectum cancers (+28.6%) and breast cancers (+20.2%) were observed. On the other hand, the largest decline for AAMR occurred for chronic obstructive pulmonary diseases (COPD) deaths (−84.2%), road traffic injuries (−38.6%), and diabetes mellitus deaths (−14.1%). Although the number of deaths was higher in 2019 than in 2013, AAMR for trachea, bronchus and lung cancers (−4.5%) and nephritis and nephrosis (−3.2%) declined. In addition, Table 2 displays the top 10 causes of death by sex. In 2019, males showed higher death rates than females for eight causes, while females had higher death rates of breast cancer and slightly higher rates for diabetes mellitus.

**Table 1:** Changes in mortality attributed to the top 10 causes of death in Malaysia.

Cause of deaths	Total deaths				Age-adjusted mortality rates (AAMR) (per 100,000 population)			
	2013	2019	Absolute change, N	Percent change (%)	2013	2019	Absolute change in AAMR	Percent change in AAMR (%)
1. Cerebrovascular diseases (stroke)	22,158	24,001	+1843	+8.3	105.8	91.3	−14.5	−13.7
2. Ischaemic heart disease	20,428	35,704	+15,276	+74.8	91.9	129.2	+37.3	+40.6
3. Lower respiratory infection	14,278	19,687	+5409	+37.9	69.6	73.5	+3.9	+5.6
4. Road traffic injuries	10,039	7024	−3015	−30.0	35.2	21.6	−13.6	−38.6
5. Chronic obstructive pulmonary disease	8666	1848	−6818	−78.7	44.2	7.0	−37.2	−84.2
6. Diabetes mellitus	8342	8712	+370	+4.4	37.7	32.4	−5.3	−14.1
7. Trachea, bronchus and lung cancers	4355	5232	+877	+20.1	20.0	19.1	−0.9	−4.5
8. Nephritis and nephrosis	3294	4078	+784	+23.8	15.4	14.9	−0.5	−3.2
9. Colon and rectum cancers	2737	4424	+1687	+61.6	12.6	16.2	+3.6	+28.6
10. Breast cancers	2082	2984	+902	+43.3	8.4	10.1	+1.7	+20.2

**Table 2:** Mortality attributed to the top 10 causes of death by sex in Malaysia.

Cause of deaths	Total deaths				Age-adjusted mortality rates (AAMR) (per 100,000 population)			
	2013		2019		2013		2019	
	Male	Female	Male	Female	Male	Female	Male	Female
1. Cerebrovascular diseases (stroke)	11,091	11,067	12,530	11,471	105.7	104.3	96.0	86.9
2. Ischaemic heart disease	12,859	7569	22,525	13,179	112.0	70.5	160.4	97.7
3. Lower respiratory infection	6942	7336	10,712	8975	68.5	69.4	80.5	66.8
4. Road traffic injuries	8383	1656	5916	1108	57.4	12.3	35.6	7.1
5. Chronic obstructive pulmonary disease	6405	2261	1387	461	68.6	22.0	10.8	3.5
6. Diabetes mellitus	3928	4414	4266	4446	35.6	39.5	32.2	32.7
7. Trachea, bronchus and lung cancers	3004	1351	3643	1589	28.1	12.3	27.3	11.4
8. Nephritis and nephrosis	1655	1639	2041	2037	15.2	15.4	15.1	14.8
9. Colon and rectum cancers	1597	1140	2593	1831	15.0	10.3	19.3	13.3
10. Breast cancers	3	2079	7	2977	0.0	16.9	0.0	20.1

## Discussion

The leading cause of death in Malaysia in 2019 was ischaemic heart disease, showing a significant increase from 2013. This increase is attributed to heightened risk factors, such as obesity, diabetes, hypertension, and high cholesterol, which collectively contribute to the growing burden of heart disease. The National Health and Morbidity Survey (NHMS) reported a rise in the prevalence of obesity and diabetes from 2011 to 2019.^5^ Also, the aging population in Malaysia is driving an uptick in heart disease deaths among older adults, constituting approximately 29.5% of deaths in 2018.^6^ The persistent prevalence of risk factors signals an impending increase in heart disease burden, necessitating urgent measures to mitigate exposure. There is a pressing need to enhance and expand prevention programs and strategies.

Conversely, the findings revealed a decline in mortality rates for CVD. One possible explanation for this contradiction could be the improvements in medical treatments and interventions for CVD,^7^ leading to a decrease mortality rates despite the prevalence of risk factors. Our analysis of mortality rates, based on data from the Malaysian population for each year of the study period, revealed a slight increase in deaths attributed to CVD. However, the increase was paralleled by a gradual rise in the population over the same period. As a result, the mortality rates were proportionally reduced, highlighting the complex interplay between population growth and mortality trends. Given these complexities, these results should be interpreted with caution.

Cancer mortality rates in Malaysia have witnessed an increase, notably for colon and rectum cancers, and breast cancers. According to the National Cancer Registry, breast and colorectal cancers were the most prevalent, comprising 19% and 14%, respectively.^8^ The report highlighted an increase in female breast cancer and male colorectal cancers from 2008 to 2016, contributing to the overall rise in cancer-related deaths. Conversely, lung cancers exhibited a reduction, leading to a decline in lung cancer death rates.

During the study period, the most substantial decline in AAMR was observed for COPD, reflecting long-term declines in death rates for COPD. Studies conducted in Sweden have shown that the decrease in prevalence of COPD follows a decrease in smoking rates.^9^ Similarly in Malaysia, the progress is attributed, in part, to a decrease in smoking among adults, as reported by the NHMS, which saw a decline from 24.8% in 1996 to 21.3% in 2019. This reduction in smoking prevalence may have contributed to the decline in COPD related deaths. Continued efforts to prevent tobacco use initiation and promote smoking cessation are crucial, given tobacco's significant role in COPD development. The implementation of smoking bans at all eateries in Malaysia suggests substantial potential for reducing the prevalence of current smoking. Additionally, it is imperative to recognize that tobacco use also serve as a significant risk factor for ischemic heart disease and CVD. It is also essential to consider the effects of tobacco use in overall cardiovascular health. Thus, considering tobacco's broader impact on cardiovascular health alongside COPD is crucial for developing effective strategies to address tobacco-related mortality.

Men generally exhibit higher mortality rates than women across most causes of death. This is often because men are more likely to engage in risky behaviours such as smoking and excessive alcohol consumption, which can lead to chronic diseases and increase mortality.^6^ Additionally, social and cultural norms may cause men to delay seeking medical attention until their condition worsens. Understanding these factors is crucial for developing targeted interventions to address health inequalities and improve overall population health.

In conclusion, our study reveals significant trends in mortality rates and disease burden in Malaysia. The prominence of non-communicable diseases, such as heart disease and cancer, as the leading cause of death underscores the urgent need for public health policies prioritizing non-communicable diseases prevention and control of these conditions and their risk factors. This includes the promotion of healthier lifestyles, the implementation of screening programs and ensuring access to high-quality healthcare services. The study's findings have broad implications for the economy, society, and public health, necessitating comprehensive strategies to effectively address these health challenges.

## Contributors

WFK conceptualised the study, analysed and interpreted the results, and wrote the original draft. HYY conceptualised the study and contributed critically reviewing the manuscript. All authors contributed to the article and approved the submitted version.

## Data sharing statement

For data protection purposes, the data used in this study cannot be accessed publicly. However, it can be obtained from the Department of Statistics, Malaysia upon reasonable request and with the necessary permissions.

## Declaration of interests

The authors declare no competing interests.
